# Chatbots to Improve Sexual and Reproductive Health: Realist Synthesis

**DOI:** 10.2196/46761

**Published:** 2023-08-09

**Authors:** Rhiana Mills, Emily Rose Mangone, Neal Lesh, Diwakar Mohan, Paula Baraitser

**Affiliations:** 1 SH24 London United Kingdom; 2 The Bill & Melinda Gates Foundation Seattle, WA United States; 3 Dimagi Cambridge, MA United States; 4 Department of International Health Johns Hopkins Bloomberg School of Public Health Baltimore, MD United States

**Keywords:** chatbot, sexual and reproductive health, realist synthesis, social networks, service networks, disclosure, artificial intelligence, sexual, reproductive, social media, counseling, treatment, development, theory, digital device, device

## Abstract

**Background:**

Digital technologies may improve sexual and reproductive health (SRH) across diverse settings. Chatbots are computer programs designed to simulate human conversation, and there is a growing interest in the potential for chatbots to provide responsive and accurate information, counseling, linkages to products and services, or a companion on an SRH journey.

**Objective:**

This review aimed to identify assumptions about the value of chatbots for SRH and collate the evidence to support them.

**Methods:**

We used a realist approach that starts with an initial program theory and generates causal explanations in the form of context, mechanism, and outcome configurations to test and develop that theory. We generated our program theory, drawing on the expertise of the research team, and then searched the literature to add depth and develop this theory with evidence.

**Results:**

The evidence supports our program theory, which suggests that chatbots are a promising intervention for SRH information and service delivery. This is because chatbots offer anonymous and nonjudgmental interactions that encourage disclosure of personal information, provide complex information in a responsive and conversational tone that increases understanding, link to SRH conversations within web-based and offline social networks, provide immediate support or service provision 24/7 by automating some tasks, and provide the potential to develop long-term relationships with users who return over time. However, chatbots may be less valuable where people find any conversation about SRH (even with a chatbot) stigmatizing, for those who lack confidential access to digital devices, where conversations do not feel natural, and where chatbots are developed as stand-alone interventions without reference to service contexts.

**Conclusions:**

Chatbots in SRH could be developed further to automate simple tasks and support service delivery. They should prioritize achieving an authentic conversational tone, which could be developed to facilitate content sharing in social networks, should support long-term relationship building with their users, and should be integrated into wider service networks.

## Introduction

### Background

A chatbot (or conversational agent) is a computer program that is designed to simulate conversation with human users. Chatbots are increasingly used in service and retail sectors [1-3] where they offer reduced reliance on human agents, 24/7 availability, and the ability to respond to large numbers of questions quickly. A similar, but slower, rise in chatbot use in education and health has also been documented [1] particularly within mental health care where chatbots provide cognitive behavioral therapy and support self-help for stress, anxiety, and difficulty sleeping [4-6].

Chatbots have been classified as “task (transaction) orientated” or “conversation-orientated” [7]. A task-orientated chatbot is designed to provide options to solve a specific problem, for example, offering customers a menu of services, whereas a conversation-orientated chatbot is designed to generate a relationship that may continue over time. They vary in their complexity from rule-based chatbots that ask users to select from a list of prewritten queries and return an answer from a pool of predetermined responses to artificial intelligence–driven models that use natural language processing to understand user queries, inputted using free text, and generate original responses. Hybrid chatbots can use elements of both approaches. They can be stand-alone interventions or integrated into mobile apps, websites, texting, smart technologies, and virtual reality sites. User input is usually in text or speech form, while the output generated by the chatbot can be written, spoken, or visual. They differ from searching the web because the responses are often conversational, and they provide a single answer with no need for assessment and filtering of many possible answers [8].

There is a small but rapidly expanding literature on chatbot design, the user experience of chatbots, and the outcomes of chatbot use. Several recent reviews inform chatbot design [9,10], the development of service chatbots [11], and the human elements of chatbot interaction [3,7].

### Chatbots to Improve Sexual and Reproductive Health

Many people still experience poor sexual and reproductive health (SRH). Globally, in 2022, 164 million women reported an unmet need for contraception, and 4000 people became infected with HIV every day [12,13]. There is some evidence to suggest that digital technologies, in general, may improve SRH, with early indications of effectiveness for improving knowledge; influencing attitudes, beliefs, and expectations; and increasing self-efficacy in support of healthy behaviors [14-16]. In this context, there is a growing interest in the potential of chatbots to deliver SRH information and services, and a growing number of chatbots are being developed in this field. Initial research on chatbots suggests low to moderate acceptability [17,18] with chatbots perceived as useful for providing automated and anonymous SRH information but as unsuitable for use in matters requiring empathy [18]. There is very little evidence on the efficacy of chatbots in improving SRH outcomes [19]. To support innovation in this field, we conducted a realist review of the literature on chatbots for SRH. We used international best practice guidelines to identify features of high-quality SRH information and services [20] and then used expert knowledge within our stakeholder group to identify where chatbots might support SRH delivery. We then reviewed the literature to develop and test these assumptions.

### Realist Synthesis

Realist synthesis is a theory-driven approach to understanding contextual influences on whether, why, and how interventions might work [21]. It starts by making explicit the underlying assumptions about how an intervention is intended to work by developing a program theory that sets out the stages of the intervention and the assumptions that underpin each stage. Empirical evidence is then collected for each stage of the program theory and is used to modify and adapt it. The results of the review are combined to explain the relationship between the context in which the intervention is applied, the mechanisms by which it works, and the outcomes that are produced. The aim is to enable decision makers to reach a deeper understanding of the intervention and how it can be made to work most effectively [22].

## Methods

### Location of Existing Theories

We followed RAMESES (Realist and Meta-Narrative Evidence Syntheses: Evolving Standards) guidance on conducting a realist review throughout the review process [21]. A realist approach to understanding interventions, in this case, “chatbots to improve SRH,” proposes that any intervention is underpinned by 1 or more theories that may be implicit or explicit. An example of a theory that is implicit in many chatbot interventions may be, “users value the anonymity offered by chatbots.” These theories set out how and why the designers anticipate their intervention will work. In a realist review, this understanding is captured via an initial program theory that summarizes these assumptions. The assumptions are then tested to understand the evidence that underpins the theory and to develop and modify it in response.

**Figure 1:**
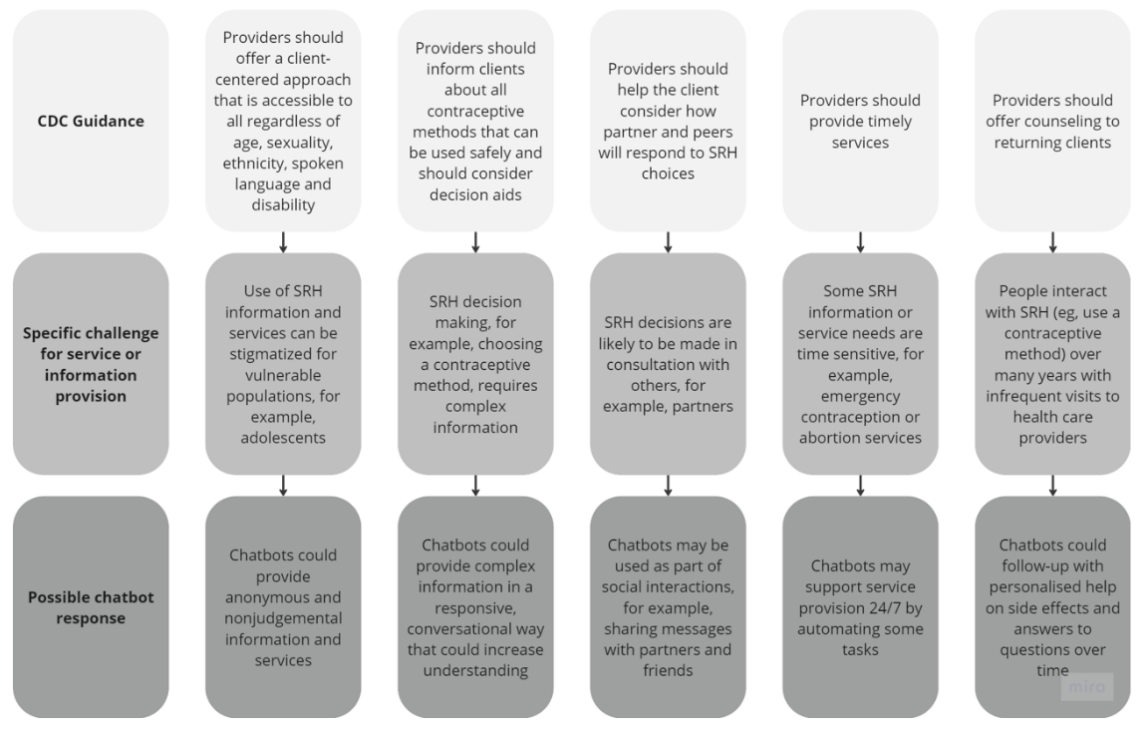
Initial program theory. CDC: Centers for Disease Control and Prevention; SRH: sexual and reproductive health.

The initial program theory was developed through 5 iterations by authors RM and PB collaboratively and discussed regularly with all authors. We started from the US Centers for Disease Control and Prevention and the US Office of Population Affairs guidance on the provision of quality family planning (and related) services [20]. We reviewed each recommendation for high-quality practice set out in this guidance and identified the service provision challenges that underpinned each one. For example, 1 recommendation suggests that providers should offer services that are accessible to all regardless of age, gender, and race. One service provision challenge that underpins this is the need to provide services that do not stigmatize users on the basis of these characteristics. For each recommendation and the challenges that underpinned it, we considered how chatbots could help. Through this process, we generated a program theory that identified where chatbots might add value to SRH service provision (Figure 1).

### Search for Evidence and Document Selection

Searching for relevant evidence within a realist review includes the following stages:

- A background search to get a feel for the literature
- Searches progressively focusing in, as the program theory develops
- A search for specific evidence to test each element of the program theory
- A final search once the synthesis is almost complete to sense check-specific findings

Our early searches performed by RM were broad and subsequently refined as our program theory developed. One database search was carried out in June 2022 and the other in December 2022, the latter search strategy is shown in Textbox 1. The search carried out in December 2022 shows a more refined search strategy that benefited from learning about chatbots from the development of the program theory. For database-specific search terms used, see Multimedia Appendix 1.

To identify gray literature sources, Google was searched using search terms “sexual health” and “chatbot,” “reproductive health” and “chatbot,” and “family planning” and chatbot. Both Google and Google Scholar were searched. Although Google search engine results are less replicable, we used this to ensure that our search for gray literature sources was as broad as possible. The first 100 hits of this Google search were screened for eligibility and included in the review based on the inclusion criteria listed in Textbox 2. Additional searches were also carried out by RM and PB to add depth to areas of interest arising from the program theory in both SRH and other fields. These theory-driven searches included, “chatbot and conversation,” “chatbot and empathy or emotion,” “chatbot and disclosure,” and “chatbots and social networks or community.” We conducted a final search in December 2022 to identify any new materials. When a potentially relevant source was identified, it was screened and assessed for eligibility using the inclusion criteria (Textbox 2).

**Searches carried out in December 2022.**

**Databases searched**

MEDLINE, Embase, Emcare, PubMed, Science Direct, Cochrane Library, Scopus, and Google Scholar

**Search terms**

“chat bot*” or chat-bot* or chatbot* or “chatter bot*” or chatterbot* or “talk bot*” or talkbot* or talk-bot* or “interactive agent*” or “conversational agent*” or “artificial conversation* entit*” or “artificial intelligence” or AI or “human computer interaction” or

“intelligent agent*” or

“chat agent*” or “relational agent*” or

“virtual agent*” or

“virtual assistant*” or

“virtual coach”

AND

“sexual and reproductive health” or “reproductive and sexual health” or “sexual health” or “reproductive health” or “sexually transmitted infection*” or STI or STIs or “sexually transmitted disease” or STD or STDs or HIV of “human immunodeficiency virus” or chlamydia or gonorrhea or herpes or “herpes genitalis” or HPV or “human papillomavirus” or syphilis or condom or “cervical cancer” or “cervical screen” or “pap* test” or antenatal or prenatal or postnatal or perinatal or pregnan* or maternal or gynae* or birth or caesarean

AND

Contracept* or “family planning” or LARC or “long acting reversible contraceptive” or “pill” or COC or POP or “progesterone only pill” or “combined oral contraception” or “inter-uterine device” or IUD or “inter-uterine system” or IUS or coil or “hormonal coil” or “copper coil” or “contracept* implant” or “injectable contracept*” or “self-injectable contracept* or “depo-provera” or “sayana Press” or “contraceptive decision making” or “family planning decision making”

**Inclusion and exclusion criteria.**

**Inclusion criteria**

- The paper must be published between 2010 and 2022.
- The intervention must include a chatbot (Chatbot or conversational agent is defined as a computer program that is designed to simulate conversation with human users).
- The intervention must aim to address an element of sexual or reproductive health (we defined sexual and reproductive health (SRH) broadly to include contraception, maternal health, diagnosis, and treatment of sexually transmitted infections).

**Exclusion criteria**

- The paper is not published between 2010 and 2022.
- The intervention does not include a chatbot (Chatbot or conversational agent is defined as a computer program that is designed to simulate conversation with human users).
- The intervention does not aim to address an element of SRH (as defined above).

### Quality Appraisal

All included papers were assessed for relevance (their ability to develop or test elements of the program theory) and for rigor (whether the methods of data collection and analysis are robust) by RM (see Multimedia Appendix 2). PB reviewed a randomly selected sample of the papers to check for agreement on quality assessment. Where appropriate the CASP Cohort Study Appraisal Tool, the CASP RCT Study Appraisal Tool [23], the CASP Qualitative Study Appraisal Tool [24], and the AACODS (Authority, Accuracy, Coverage, Objectivity, Date, Significance) checklist for gray literature [25] were used to guide the critical appraisal process.

### Data Extraction

Following the RAMESES guidelines for realist review, we were not prescriptive about what data should be extracted. However, we aimed to demonstrate the link between the research question and the category of data extracted throughout.

No uniform data set was extracted from each paper, rather the data (verbatim sections of text) from each paper that were relevant to each section of the program theory were grouped together in spreadsheets. As our theory evolved, we identified new data needs and revisited the same study to extract different findings.

### Stakeholder Group

A core group of experts (ERM, NL, DM) met every 2 weeks to review emerging findings which were progressively included in the program theory and the developing list of context, mechanism, outcome configurations (CMOCs), and the research agenda.

### Data Analysis and Synthesis

The final selected papers were read and reread by RM and a sample was read by PB. Findings were summarized in spreadsheets that contained information on key relevant findings from each paper, and grouped according to the program theory. RM and PB then developed CMOCs for each element of the program theory and developed the program theory in response.

A realist logic of analysis uses data to produce causal explanations for outcomes that occur within a program theory in the form of CMOCs. A CMOC is a proposition that explains what element of an initiative works, for whom, and in what circumstances and is the primary way of reporting findings within a realist review. Within a CMOC, the causal claim being made is that when a particular context is present, it “triggers” or “activates” a particular mechanism, which causes a particular outcome. Mechanisms are hidden causal processes that are context-sensitive and are usually inferred based on interpretations of the data. Data to inform our interpretation of the relationships between contexts, mechanisms, and outcomes were sought within and across documents so that mechanisms inferred from one document helped explain the way contexts influenced outcomes in a different document. During our analysis, we used interpretive cross-case comparison to understand and explain how and why reported outcomes have occurred.

Where there was limited evidence within the papers on SRH chatbots identified, we completed individual searches of the literature on chatbots outside SRH to locate evidence from other areas of study that was relevant to our areas of interest.

## Results

### Overview

**Figure 2:**
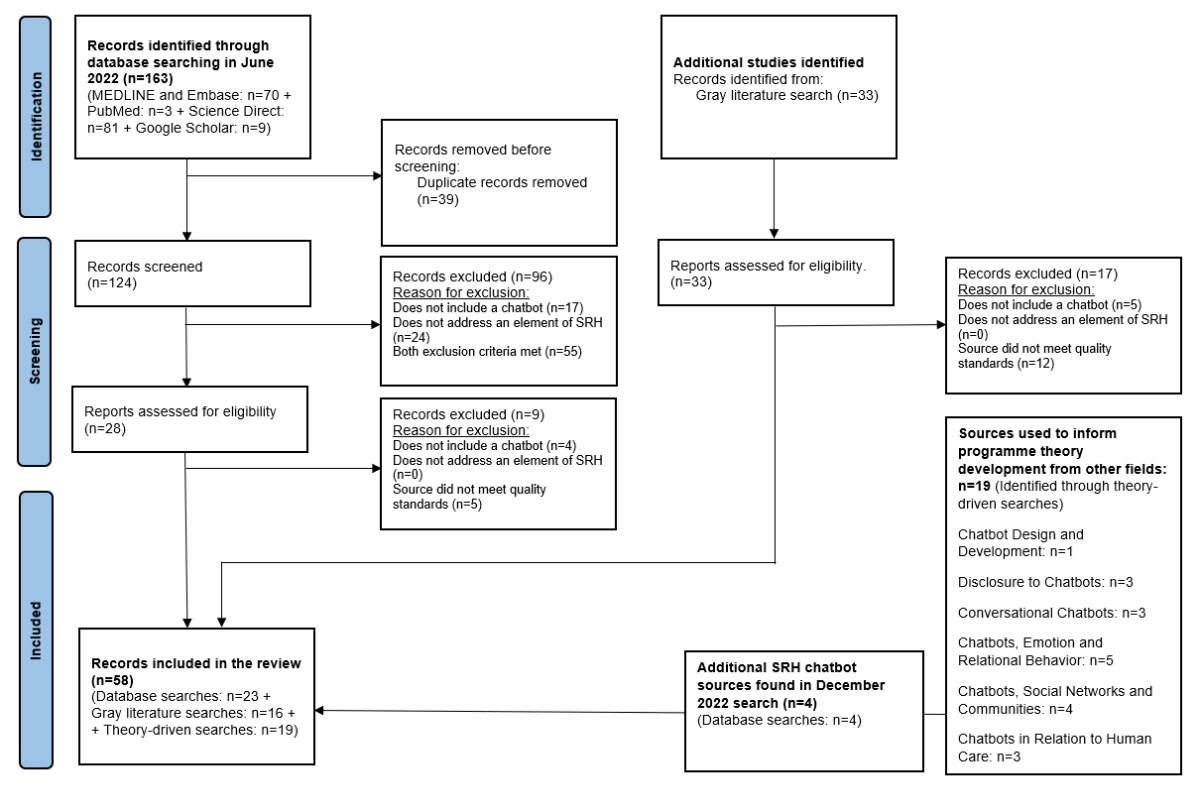
PRISMA (Preferred Reporting Items for Systematic Reviews and Meta-Analyses) 2020 flow diagram for new systematic reviews, which included searches of databases and other sources (adapted from Page et al [26]). SRH: sexual and reproductive health.

Through database searches in June 2022, 163 sources were identified (Figure 2). References were imported into Mendeley, and duplicate sources were removed (n=39). Abstracts were screened in accordance with the inclusion and exclusion criteria (Textbox 2). After this process, 28 sources remained; the full text of these documents was rescreened, and 19 sources from database searches were included in the review. Gray literature searches identified 33 sources that were screened for eligibility, with 16 gray literature sources included in the review. Four additional sources were found in a database search carried out in December 2022. Theory-driven searches aimed at developing the program theory identified 19 sources outside of SRH.

### Characteristics of Included Papers

We identified 39 SRH sources through database and gray literature searches. This included 19 peer-reviewed original research papers, 3 peer-reviewed narrative reviews, 11 website articles, 2 technical reports published on the internet, 4 other types of gray literature, a letter, 2 theses, and a short report. Where sources reported on the geographical context where chatbots were implemented, 15 were implemented in high-income countries, with the majority in the United States (n=9) and United Kingdom (n=4) and 2 implemented in Japan. In low-income countries, 22 chatbots were implemented, the majority in African countries (n=15), including Kenya (n=7), South Africa (n=2), the Democratic Republic of the Congo (n=1), Uganda (n=2), and Nigeria (n=1). Chatbots were implemented in other low-income countries, including India (n=5), Bangladesh (n=1), and Mongolia (n=1). To inform the development of the program theory, 19 papers from other disciplines were also included. Three papers examine user disclosure of information to chatbots; 3 seek to understand the conversational aspects of chatbots; and 5 explore chatbots, emotionality, empathy, and human-bot relational behavior. Four papers explore social networks or nondyadic chatbots and their interactions with communities. Three papers report on chatbots and their relationship to wider service networks. One paper explores chatbot design and development.

Multimedia Appendix 2 describes all included papers (n=58) with author, date, title, country of research, and source type, and summarizes data on study design and the reviewer’s assessment of rigor, relevance, and plausibility.

### Realist Synthesis: Mapping Evidence Into the Initial Program Theory

The realist synthesis seeks to map the evidence base onto the initial program theory to interrogate the assumptions it makes and add depth and detail to the theory. The following subheadings are taken from the “Possible Chatbot Response” section of the initial program theory (see Figure 1).

### Chatbots Could Provide Anonymous and Nonjudgmental SRH Information and Services

The literature on chatbots for SRH suggests that people value the anonymous and nonjudgmental space that chatbots offer for SRH discussions, particularly in contexts where SRH is stigmatized, or perceived as stigmatized, and for groups that face or perceive SRH stigma [17,18,27-38]. The literature that supports stigma as a barrier, across many contexts, and the evidence on chatbots as a response to this, included high-income countries (n=5) and low-income countries (n=7). The value of chatbots as a strategy to offer nonstigmatizing services is supported by substantial evidence from outside SRH settings, mainly from mental health care, which shows that people are more likely to disclose sensitive information to chatbots than to humans [39-42]. It seems that both perceived anonymity [43] and reduced fear of negative or judgmental responses are important for disclosure [40].

The SRH literature suggests that the anonymity or confidentiality of chatbot use requires access to a private digital device, and those without this access will be excluded from this benefit [37,38] (Table 1). Where people experience conversations about SRH as taboo, this may also apply to conversations with chatbots [35,37]. Adolescent girls in India were much less likely to engage with a chatbot aimed at adolescents of all genders than boys; it is suggested that this may be because female users in India face gender disparities in mobile device ownership and low digital media literacy [37]. Adolescent girls in this context may also be less comfortable about openly discussing SRH and may hold higher levels of privacy concerns due to taboos concerning SRH for girls [37].

**Table 1:** Context, mechanism, outcome configurations (CMOCs) regarding chatbot delivery of anonymous and nonjudgmental SRH information and services.

CMOCs	Studies
In contexts where conversations about SRH^a^ generate stigma and embarrassment (C), people may engage with chatbots (O) because chatbots are nonjudgmental and anonymous (M)	[17,18,27-33,35-38,44-48]
In contexts where conversations about SRH are taboo (C), some populations may not engage with SRH chatbots (O), because discussing SRH even through an anonymous medium remains stigmatizing (M)	[35,37]
Where chatbots assure users that their information will be kept anonymous and their privacy will be maintained (C), users may engage with the chatbot (O), because their concerns have been addressed (M)	[33,49-51]
Where users do not have access to a private digital device (C), users are not afforded anonymity with chatbot use (O), because they cannot assure that their interaction with the chatbot will not be seen by other users of the device (M)	[37,38,52,53]

^a^SRH: sexual and reproductive health.

### Chatbots Could Provide Complex Information in a Responsive and Conversational Way

Maintaining SRH requires access to and understanding of complex information including information on the different contraceptive methods and how to use them, sexually transmitted diseases and how to test for them, and HIV prevention such as the use of pre-exposure prophylaxis. Complex information may be better understood if delivered in a conversational format.

We found the definition of “conversation” proposed by Zamani et al [8], in their monograph on conversational information seeking, useful; “a sequence of interactions between 2 or more participants including humans and machines as a form of interactional communication with the goal of information exchange” [8]. Key features of conversational information are that it is delivered in short segments, there is an opportunity to check understanding or ask clarification questions and the tone of voice is engaging [54]. There is also evidence that information presented in a conversational form is easier to understand and more engaging, particularly for those with low health literacy [54,55].

A dialogical structure allows complex information to be conveyed in segments or “chunks” rather than long passages of text, making complex information more digestible to users and easier to understand [56]. Sharing information in this way may be valued over using search engines as users do not have to search, appraise sources, or pick out answers from longer passages of text [17,18,29,30,35,49,57]. Chatbots may also check user understanding and well-being at various points in the conversation [18,30,35,38,50,58]. This allows users to evaluate whether their needs are being met by the chatbot and may feel like a more authentic conversational flow.

The extent to which the conversations generated feel “human” is important. SRH chatbots vary widely in their conversational ability, from those that offer menus of questions that are chosen by typing a number, to chatbots that interpret free text questions and generate personalized responses. Evidence from chatbots both in SRH and other areas of health care shows that engaging chatbots use conversational strategies such as a friendly tone, demonstrating active listening (eg, paraphrasing), showing empathy, and using familiar language [3,7,9,35,37,57] validating feelings [30,52,58] prompting further questions and checking understanding [18,30,35,50,55,58].

When the language used makes chatbots feel uncanny (not quite human), like replying too quickly, misunderstanding questions, or using overly formal language, then this makes interactions less conversational and potentially distracts from understanding complex information [18,31,58]. Conversational breakdowns between humans and chatbots are common and effective repair strategies are important, for example, chatbots that acknowledge that there has been a conversational breakdown and show initiative from the chatbot to recover are preferred [7].

There are also concerns about chatbots, particularly artificial intelligence chatbots that do not rely on prewritten responses and may engage in conversation but misinterpret questions or provide inaccurate information [59] (Table 2). Incorrect answers could generate health risks where users act on inappropriate clinical advice or signposting [59,60].

**Table 2:** Context, mechanism, outcome configurations (CMOCs) regarding chatbot provision of complex information.

CMOCs	Studies
When chatbots provide access to accurate information in digestible form (C), chatbots may be preferred to search engines (O), as the chatbot can eliminate steps to search and filter web-based health information (M)	[17,18,29,30,35,48,49,57,61]
When the language cues used, make chatbots feel uncanny (not quite human), like replying too quickly, misunderstanding, or overly formal language (C), then users can disengage from connecting with the Chatbot (O), as humans are sensitive to language cues that do not “feel right” (M)	[18,31,58,62]
When chatbots interact with users by prompting further questions and checking in with them (C), users engage for longer with the chatbot (C), because interaction drives the “conversation” between the user and chatbot forward and feels more human (M)	[18,30,35,38,50,53,58]
Where chatbots repeat information, either during a single session over repeated sessions (C), users may engage with the information provided (O), because repetition reinforces understanding (M)	[28,30]
Where chatbots use language that validates users’ feelings and needs (C), this may engage users in chatbot use (O), because the chatbot offers a feeling of being understood (M)	[30,52,58]
Where chatbots give complex information on SRH topics (C), users may be able to understand the information more easily (O), because the information is given in a dialogical structure that shares information in short segments of “chunks” (O)	[54,56,62,63]

### Chatbots May Mimic SRH Information Sharing as Part of Social Interactions, for Example, Sharing Messages With Partners and Peers

Most people obtain SRH information through conversation with friends and family [64-68], and this method of seeking and understanding SRH information is familiar. Chatbots that adopt engaging and appropriate human behaviors, which were discussed in the section above, mimic familiar conversations about SRH. Users may build relationships with chatbots, human agents, and peers within a single hybrid system, so chatbots are one element of a complex and networked set of relationships [9]. As part of this network, text from automated SRH interventions may be shared with partners and used as a basis for SRH conversations [37], for example, informing partners about the diagnosis of a sexually transmitted infection and negotiating testing [69].

As well as mimicking conversations with family and peers, chatbots can also model safe and open conversations about SRH, potentially affording users SRH communication skills. Chatbot developers may invest in content that is sex positive, inclusive of all expressions of gender and sexuality, and delivered in an open and nonjudgmental tone of voice. Where this content and tone are modeled by chatbots, it can be shared as alternatives to sex-negative, noninclusive, stigmatizing, and judgmental SRH messages.

Understanding how chatbots operate in social networks is being explored through chatbots that interact with web-based communities, including web-based health communities [70-73]. These chatbots initiate and support interactions within groups where the chatbot intervention is seen by everyone and chatbots must navigate complex conversational skills such as turn-taking [70]. Early research in this field has used a “community” of humans to train a chatbot to interact in a particular social context over time, where the chatbot learns socially appropriate responses from the community and develops to become recognized as a legitimate member of that group.

Reviews of the importance of personas in health care chatbots suggest that the look and feel of web-based health assistants may affect user experience, although the perception of a social presence may be more important than an avatar [3,74] (Table 3). Specific personality types for chatbots in health settings have been described including a supportive, coaching-type personality and a more formal, health care professional [9].

The relational nature of chatbots may have some benefits but may also work to disappoint users. Users may form a bond with the chatbot, personify and respond to the chatbot with empathy, as if it were a human [35,37,38]. In turn, users may feel disappointed in the limitations of the human-bot relationship, by wanting greater intimacy [37] or greater conversational width and depth [35].

**Table 3:** Context, mechanism, outcome configurations (CMOCs) regarding chatbots mimicking information sharing as part of social interactions.

CMOCs	Studies
When chatbots reference context-specific SRH^a^ norms (such as SRH information seeking from peers or relatives) (C), then they may be engaging (O), because they feel familiar and relevant (M)	[35,38]
Where chatbots use emojis in a context-appropriate way (C), users enjoy the use of emojis (O), because they mimic conversations with friends and family (M)	[30,53,58,75]
Where users have formed a relationship with a chatbot (C), they may interact with it as if it were human including displays of empathy and inappropriate behavior (O), because the user may have personified the chatbot (M)	[35,37,38]
Where users have bonded with a chatbot (C), users may be disappointed by the limitations of a relationship with a chatbot (O), because chatbots cannot offer the depth of a human relationship (M)	[35,37]
When a chatbot persona resembles someone users feel comfortable discussing SRH with (C), then users may use the chatbot (O), because it relates to other experiences of positive SRH conversations (M)	[37,38,57]

^a^SRH: sexual and reproductive health.

### Chatbots May Support Service Provision 24/7 by Automating Some Tasks

The SRH literature suggests that users value transactional chatbots that offer SRH information or services quickly and efficiently since users seek information reactively in response to real-time SRH concerns that arise [17,18,29,30,35,49,57] (Table 4). Some elements of SRH provision are time sensitive such as access to emergency contraception, ongoing contraception (eg, when someone runs out of contraceptive pills), or postexposure prophylaxis for HIV. In these situations, chatbots may provide a rapid and timely assessment (eg, is emergency contraception needed) and signposting to wider services.

**Table 4:** Context, mechanism, outcome configurations (CMOCs) regarding chatbot provision of 24/7 services.

CMOCs	Studies
When chatbots offer 24-hour access to SRH^a^ information (C), then users may find this convenient (O), because access to SRH information is not constrained by service opening times (M)	[18,31,35,38,48,58]
When chatbots provide immediate access to accurate information (C), users may stay engaged (O), as some users use chatbots in times of immediate need that require a fast response (M)	[17,18,29,30,35,49,57]

^a^SRH: sexual and reproductive health.

### Chatbots Could Follow-Up With Personalized Help on Side Effects and Answers to Questions Over Time

Chatbots may be designed to generate the possibility of future interactions or to solve specific questions on a one-off basis. Users’ engagement with chatbots may change over time, with a “testing phase” being reported by developers of a chatbot that engaged with a web-based gaming community [70]. In initial interactions, group members tested the boundaries of interacting with the chatbot, such as seeing whether it would respond to rude comments or expletives [70].

Research on chatbot development suggests that the temporality of interactions should be built into chatbot architecture and identifies 3 temporal chatbot archetypes: ad hoc supporter, temporal advisor, and persistent companion [76] (Table 5). Persistent companion chatbots, for example, a chatbot for preconception behavior change [77], stimulate longer engagement and repeated conversations with goal setting and progress reporting and the likelihood of behavior change increases with repeated interactions as information is reiterated and reinforced [55,77]. Re-engaging users to return to ad hoc supporter chatbots can be a difficult task [49]. Users may return organically to ad hoc supporter chatbots that are easy to use, provide quick and accurate responses, and are trusted by the user [37,38,75].

**Table 5:** Context, mechanism, outcome configurations (CMOCs) regarding chatbot provision of personalized follow up.

CMOCs	Studies
Where chatbots set goals for completion over time (C) then users may return to them over time (O) as they value recording their progress (M)	[77,78]
Where chatbots provide quick and accurate responses that are trusted by the user (C) then users may return to them over time (O) because they are convenient to use	[37,38,75]

### Chatbots Support Users Best When They Link Users to a Wider SRH Service Network

Although not anticipated in the initial program theory, the importance of chatbots as signposters and referrers came out strongly in the literature [18,34,36,44,46,50] (Table 6). Chatbots may link to face-to-face SRH services, SRH helplines, and web-based human agents [57,79]. Chatbots can be useful to answer user questions, help with taking medical histories, help users fill in forms, triage, and signpost patients to the appropriate face-to-face care. The wider literature on chatbots, from pandemic health to mental health suggests that chatbots work best when they augment face-to-face care [80-82]. Service chatbots benefit from integration into a wider customer service network, where a chatbot can support links to related services, when it does not have the functionality to meet a customer’s needs [83]. “Actionability” has been identified as a key “affordance” of chatbots where users take action as a result of their conversation such as calling a helpline suggested by a chatbot [37].

**Table 6:** Context, mechanism, outcome configurations (CMOCs) regarding chatbot linkage to wider SRH^a^ service networks.

CMOCs	Studies
Where chatbots are used alongside human care (C), users find chatbots more acceptable (O), because users do not find chatbots appropriate as a complete alternative to human care (M)	[17,28,33,58]
Where chatbots are integrated into wider service networks (C), users’ need for SRH services is better met (O), because users are signposted and supported in seeking other SRH services (M)	[18,34,36,44,46,50,51,84]

^a^SRH: sexual and reproductive health.

## Discussion

**Figure 3:**
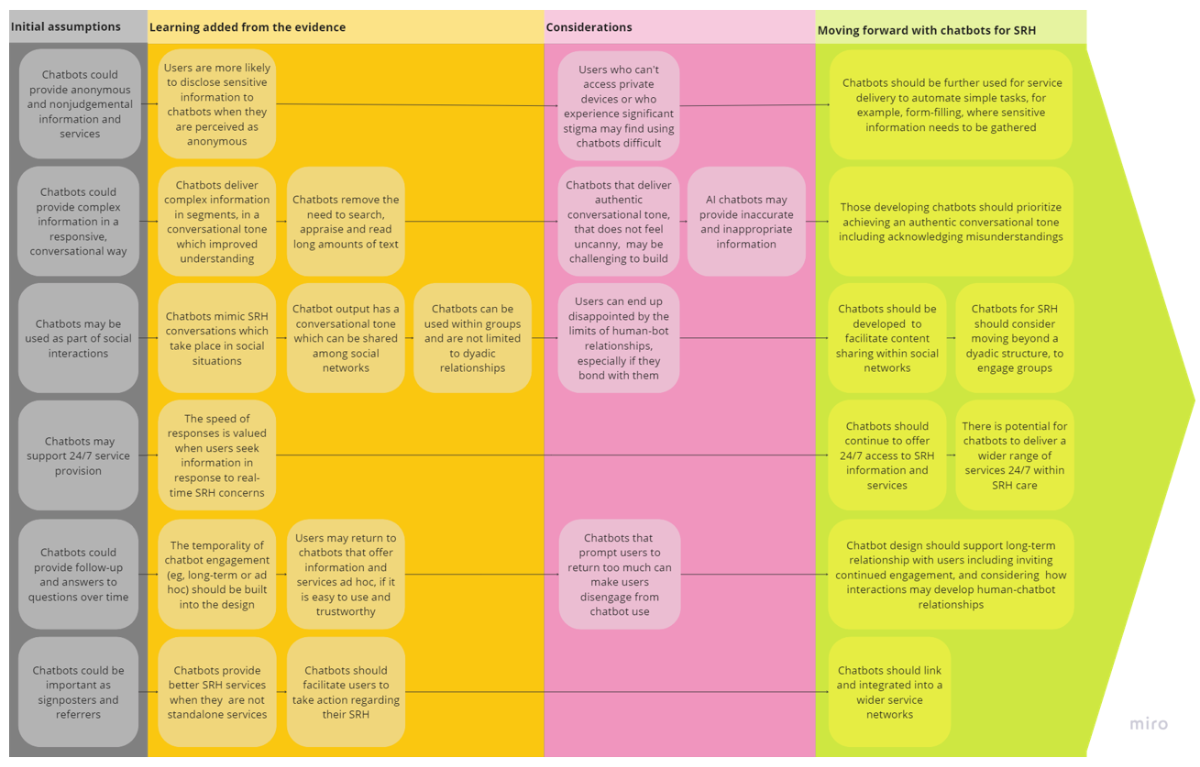
Final program theory. AI: artificial intelligence; SRH: sexual and reproductive health.

We found evidence to support all of the assumptions about the potential value of chatbots to support specific elements of SRH provision proposed in our initial program theory. Consideration of the evidence for each enabled us to develop and refine this theory further as shown in Figure 3.

### Principal Findings

The initial program theory proposed that users value the anonymity and lack of human judgment that chatbots afford. Chatbots could provide complex information in a responsive and conversational way that could increase understanding. Chatbots may be used as part of social interactions, for example sharing messages with partners and friends. Chatbots may support service provision 24/7 by automating some tasks, and chatbots could follow up with personalized help on side effects and answers to questions over time.

The evidence supports these propositions suggesting that people are more likely to disclose sensitive information to chatbots and that engaging with a chatbot may be less embarrassing, less stigmatizing, and more private than other SRH services, although for those facing serious stigma or who lack access to a private digital device, using a chatbot may still be difficult. There is evidence to suggest that the conversational structure that chatbots use delivers information that is digestible, engaging, and accessible, and linked to web-based and offline social networks, although the reality of the limits of a human-bot relationship can disappoint users and lead to disengagement. The evidence suggests that the constant availability of chatbots means that they can provide information and signposting quickly and may offer immediate care and encourage users to return to the chatbot to learn additional information and work toward goals. Finally, the benefit of chatbot integration into wider service networks emerged from the evidence. 

### Implications

The evidence suggests that chatbots are a promising intervention for SRH information and service delivery. Chatbots for SRH should be able to hold authentic conversations and transition users to human agents when the conversation goes beyond the scope of the chatbot or when complex health or safeguarding issues are raised. There is potential for chatbots to be integrated into wider service and social networks; this would require chatbot development that references the possibilities for sharing of information provided by chatbots outside a dyadic interaction and the development of chatbots that interact as part of group conversations. Subsequent chatbot design should also consider how human-chatbot interaction may change over time. Although the broader literature on chatbots in health care references their importance as agents for service delivery, this use of chatbots remains underdeveloped within the SRH space.

### Strengths and Limitations

This is the first realist review of chatbots specifically for SRH. This paper is systematic and transparent in its approach to the realist review, which was conducted in accordance with the RAMESES standards [21]. Our authorship team represents a variety of academic and technical backgrounds, ensuring divergence in our analysis, and we benefited from expert feedback from a core group of stakeholders. Limitations include our focus on publicly accessible literature, located through recognized research databases and Google. It is likely that some chatbots for SRH are never evaluated or documented in the public domain. It may be that service chatbots are particularly hidden. Searches were carried out in English only, evidence published in other languages is missing from this review.

### Conclusions

The evidence supports our program theory, which suggests that chatbots are a promising intervention for SRH information and service delivery, due to affordances specific to chatbots. Chatbots in SRH could be developed further: to automate simple tasks and support service delivery, to prioritize achieving an authentic conversational tone, to facilitate content sharing in social networks, to support long-term relationship building with their users, and to be integrated into wider service networks. These developments would advance their potential to better respond to users existing digital and social information sharing practices and the need for digestible and anonymous SRH information and signposting.

## Appendix

Multimedia Appendix 1 Database search terms.

Multimedia Appendix 2 Table of results.

## Abbreviations

CMOC: context, mechanism, outcome configuration
RAMESES: Realist and Meta-Narrative Evidence Syntheses: Evolving Standards
SRH: sexual and reproductive health
