# Supplementary material for: Chatbots to Improve Sexual and Reproductive Health: Realist Synthesis
Source: J Med Internet Res. 2023 Aug 9;25:e46761. doi: 10.2196/46761 (PMC10448286; doi:10.2196/46761)
Supplement: Multimedia Appendix 1 [file jmir_v25i1e46761_app1.docx]

Search String Medline

1. ("chat bot*" or chat-bot* or chatbot* or "chatter bot*" or chatterbot* or "talk bot*" or talkbot* or talk-bot* or "interactive agent*" or "conversational agent*" or "artificial conversation* entit*" or "artificial intelligence" or AI or "human computer interaction" or "intelligent agent*" or "chat agent*" or "relational agent*" or "virtual agent*" or "virtual assistant*" or "virtual coach tw").af.

2. ("sexual and reproductive health" or "reproductive and sexual health" or "sexual health" or "reproductive health" or "sexually transmitted infection*" or STI or STIs or "sexually transmitted disease" or STD or STDs or HIV or “human immunodeficiency virus" or chlamydia or gonorrhoea or herpes or "herpes genitalis" or HPV or "human papillomavirus" or syphilis or condom or "cervical cancer" or "cervical screen" or "pap* test" or antenatal or prenatal or postnatal or perinatal or pregnan* or maternal or gynae* or birth or cesarean tw).af.

3. (contracept* or "family planning" or LARC or "long-acting reversible contracept*" or "pill" or COC or POP or "progesterone only pill" or "combined oral contracept*" or "inter-uterine device" or IUS or "inter-uterine system" or IUS or coil or "hormonal coil" or "copper coil" or "contracept* implant" or "injectable contracept*" or "self-injectable contracept*" or "depo-provera" or "sayana press" or "contracept* decision making*" or "family planning decision making tw").af.

4. 1 AND 2 AND 3

Search String Embase

1. ("chat bot*" or chat-bot* or chatbot* or "chatter bot*" or chatterbot* or "talk bot*" or talkbot* or talk-bot* or "interactive agent*" or "conversational agent*" or "artificial conversation* entit*" or "artificial intelligence" or AI or "human computer interaction" or "intelligent agent*" or "chat agent*" or "relational agent*" or "virtual agent*" or "virtual assistant*" or "virtual coach tw").af.

2. ("sexual and reproductive health" or "reproductive and sexual health" or "sexual health" or "reproductive health" or "sexually transmitted infection*" or STI or STIs or "sexually transmitted disease" or STD or STDs or HIV or “human immunodeficiency virus" or chlamydia or gonorrhoea or herpes or "herpes genitalis" or HPV or "human papillomavirus" or syphilis or condom or "cervical cancer" or "cervical screen" or "pap* test" or antenatal or prenatal or postnatal or perinatal or pregnan* or maternal or gynae* or birth or cesarean tw).af.

3. (contracept* or "family planning" or LARC or "long-acting reversible contracept*" or "pill" or COC or POP or "progesterone only pill" or "combined oral contracept*" or "inter-uterine device" or IUS or "inter-uterine system" or IUS or coil or "hormonal coil" or "copper coil" or "contracept* implant" or "injectable contracept*" or "self-injectable contracept*" or "depo-provera" or "sayana press" or "contracept* decision making*" or "family planning decision making tw").af.

4. 1 AND 2 AND 3

Search String Emcare

1. ("chat bot*" or chat-bot* or chatbot* or "chatter bot*" or chatterbot* or "talk bot*" or talkbot* or talk-bot* or "interactive agent*" or "conversational agent*" or "artificial conversation* entit*" or "artificial intelligence" or AI or "human computer interaction" or "intelligent agent*" or "chat agent*" or "relational agent*" or "virtual agent*" or "virtual assistant*" or "virtual coach tw").af.

2. ("sexual and reproductive health" or "reproductive and sexual health" or "sexual health" or "reproductive health" or "sexually transmitted infection*" or STI or STIs or "sexually transmitted disease" or STD or STDs or HIV or “human immunodeficiency virus" or chlamydia or gonorrhoea or herpes or "herpes genitalis" or HPV or "human papillomavirus" or syphilis or condom or "cervical cancer" or "cervical screen" or "pap* test" or antenatal or prenatal or postnatal or perinatal or pregnan* or maternal or gynae* or birth or cesarean tw).af.

3. (contracept* or "family planning" or LARC or "long-acting reversible contracept*" or "pill" or COC or POP or "progesterone only pill" or "combined oral contracept*" or "inter-uterine device" or IUS or "inter-uterine system" or IUS or coil or "hormonal coil" or "copper coil" or "contracept* implant" or "injectable contracept*" or "self-injectable contracept*" or "depo-provera" or "sayana press" or "contracept* decision making*" or "family planning decision making tw").af.

4. 1 AND 2 AND 3

Search String PubMed

#1. ("chat bot*"[Title/Abstract] OR "chat bot*"[Title/Abstract] OR "chatbot*"[Title/Abstract] OR "chatterbot*"[Title/Abstract] OR "interactive agent*"[Title/Abstract] OR "conversational agent*"[Title/Abstract] OR "artificial intelligence"[Title/Abstract] OR "AI"[Title/Abstract] OR "human computer interaction"[Title/Abstract] OR "intelligent agent*"[Title/Abstract] OR "chat agent*"[Title/Abstract] OR "relational agent*"[Title/Abstract] OR "virtual agent*"[Title/Abstract] OR "virtual assistant*"[Title/Abstract] OR "virtual coach"[Title/Abstract])

#2. ("sexual and reproductive health"[Title/Abstract] OR "reproductive and sexual health"[Title/Abstract] OR "sexual health"[Title/Abstract] OR "reproductive health"[Title/Abstract] OR "sexually transmitted infection*"[Title/Abstract] OR "STI"[Title/Abstract] OR "STIs"[Title/Abstract] OR "sexually transmitted disease"[Title/Abstract] OR "STD"[Title/Abstract] OR "STDs"[Title/Abstract] OR "chlamydia"[Title/Abstract] OR "gonorrhoea"[Title/Abstract] OR "herpes"[Title/Abstract] OR "herpes genitalis"[Title/Abstract] OR "HPV"[Title/Abstract] OR "human papillomavirus"[Title/Abstract] OR "syphilis"[Title/Abstract] OR "condom"[Title/Abstract] OR "cervical cancer"[Title/Abstract] OR "cervical screen"[Title/Abstract] OR "pap test"[Title/Abstract] OR "antenatal"[Title/Abstract] OR "prenatal"[Title/Abstract] OR "postnatal"[Title/Abstract] OR "perinatal"[Title/Abstract] OR "pregnan*"[Title/Abstract] OR "maternal"[Title/Abstract] OR "gynae*"[Title/Abstract] OR "birth"[Title/Abstract] OR "cesarean"[Title/Abstract])

#3. ("contracept*"[Title/Abstract] OR "family planning"[Title/Abstract] OR "LARC"[Title/Abstract] OR "long acting reversible contracept*"[Title/Abstract] OR "pill"[Title/Abstract] OR "COC"[Title/Abstract] OR "POP"[Title/Abstract] OR "progesterone only pill"[Title/Abstract] OR "combined oral contracept*"[Title/Abstract] OR "IUS"[Title/Abstract] OR "coil"[Title/Abstract] OR "hormonal coil"[Title/Abstract] OR "copper coil"[Title/Abstract] OR "injectable contracept*"[Title/Abstract] OR "depo-provera"[Title/Abstract] OR "sayana press"[Title/Abstract] OR "family planning decision making"[Title/Abstract])

#4. #1 AND #2 AND #3

Search String Science Direct

(500-character search term limit)

"chat bot" or "interactive agent" or "conversational agent" or "artificial conversation entit" "chat agent" or "relational agent" or "virtual agent" or "virtual assistant" or "virtual coach” AND "sexual and reproductive health" or "sexual health" or "reproductive health" or "sexually transmitted infection" or condom or "cervical screen" or antenatal or postnatal or maternal AND contraception or "family planning" or "contraception decision making" or "family planning decision making"

Search String Cochrane Library

"chat bot*" or chat-bot* or chatbot* or "chatter bot*" or chatterbot* or "talk bot*" or talkbot* or talk-bot* or "interactive agent*" or "conversational agent*" or "artificial conversation* entit*" or "artificial intelligence" or AI or "human computer interaction" or "intelligent agent*" or "chat agent*" or "relational agent*" or "virtual agent*" or "virtual assistant*" or "virtual coach" in Title Abstract Keyword AND "sexual and reproductive health" or "reproductive and sexual health" or "sexual health" or "reproductive health" or "sexually transmitted infection*" or STI or STIs or "sexually transmitted disease" or STD or STDs or HIV or “human immunodeficiency virus" or chlamydia or gonorrhoea or herpes or "herpes genitalis" or HPV or "human papillomavirus" or syphilis or condom or "cervical cancer" or "cervical screen" or "pap* test" or antenatal or prenatal or postnatal or perinatal or pregnan* or maternal or gynae* or birth or cesarean in Title Abstract Keyword AND contracept* or "family planning" or LARC or "long-acting reversible contracept*" or "pill" or COC or POP or "progesterone only pill" or "combined oral contracept*" or "inter-uterine device" or IUS or "inter-uterine system" or IUS or coil or "hormonal coil" or "copper coil" or "contracept* implant" or "injectable contracept*" or "self-injectable contracept*" or "depo-provera" or "sayana press" or "contracept* decision making*" or "family planning decision making" in Title Abstract Keyword - (Word variations have been searched)

Search String Scopus

1. ( TITLE-ABS-KEY ( contracept* OR "family planning" OR larc OR "long-acting reversible contracept*" OR "pill" OR coc OR pop OR "progesterone only pill" OR "combined oral contracept*" OR "inter-uterine device" OR ius OR "inter-uterine system" OR ius OR coil OR "hormonal coil" OR "copper coil" OR "contracept* implant" OR "injectable contracept*" OR "self-injectable contracept*" OR "depo-provera" OR "sayana press" OR "contracept* decision making*" OR "family planning decision making" ) )

2. ( TITLE-ABS-KEY ( "sexual and reproductive health" OR "reproductive and sexual health" OR "sexual health" OR "reproductive health" OR "sexually transmitted infection*" OR sti OR stis OR "sexually transmitted disease" OR std OR stds OR hiv OR "human immunodeficiency virus" OR chlamydia OR gonorrhoea OR herpes OR "herpes genitalis" OR hpv OR "human papillomavirus" OR syphilis OR condom OR "cervical cancer" OR "cervical screen" OR "pap* test" OR antenatal OR prenatal OR postnatal OR perinatal OR pregnan* OR maternal OR gynae* OR birth OR cesarean ) )

3. ( TITLE-ABS-KEY ( "chat bot*" OR chat-bot* OR chatbot* OR "chatter bot*" OR chatterbot* OR "talk bot*" OR talkbot* OR talk-bot* OR "interactive agent*" OR "conversational agent*" OR "artificial conversation* entit*" OR "artificial intelligence" OR ai OR "human computer interaction" OR "intelligent agent*" OR "chat agent*" OR "relational agent*" OR "virtual agent*" OR "virtual assistant*" OR "virtual coach" ) )

Combine Queries 1 AND 2 AND 3

Search String Google Scholar

(Limited character search)

"chat bot" OR chatbot OR "conversational agent" AND contracept* OR “family planning”

Search String Google (gray literature search)

"chat bot" OR chatbot OR "conversational agent" AND contracept* OR “family planning”
