# Supplementary material for: Chatbots to Improve Sexual and Reproductive Health: Realist Synthesis
Source: J Med Internet Res. 2023 Aug 9;25:e46761. doi: 10.2196/46761 (PMC10448286; doi:10.2196/46761)
Supplement: Multimedia Appendix 2 [file jmir_v25i1e46761_app2.docx]

| **First Author** | **Date** | **Title** | **Source Type** | **Country** | **Study Design (if applicable)** | **Is the source relevant to the question?** | **Are the methods used appropriate (if applicable)?** | **Are the findings and/or conclusions of the source plausible?** | **Do the conclusions of the source support those of other sources?** |
| --- | --- | --- | --- | --- | --- | --- | --- | --- | --- |
| Abbas | 2022 | Online chat and chatbots to enhance mature student engagement in higher education | Peer reviewed journal article | UK | Mixed methods pilot study | Yes, this source explored the use of chatbots outside a dyadic dynamic. | Yes, multiple qualitative methods (surveys, interviews) are used to assess how online communities which include a chatbot can increase social interactions. | Yes | Yes |
| Almalki | 2020 | Health Chatbots for Fighting COVID-19: a Scoping Review | Peer reviewed journal article | N/A | Scoping review | Somewhat , this review describes applications of chatbots in a pandemic setting, including chatbot applications in relation to human care, and challenges with using chatbots. | Yes, a scoping review describes the current landscape of chatbots being used in a pandemic setting. | Yes | Yes |
| Bae Brandtzæg | 2021 | When the Social Becomes Non-Human: Young People’s Perception of Social Support in Chatbots. | Gray literature: Conference paper | Norway | Qualitative study | Yes, this paper attempts to understand how young people experience chatbots as a source of emotional and social support. | Yes, in-depth qualitative interviews elicit understanding of young peoples experiences. | Yes | Yes |
| Barnett | 2021 | Enacting ‘more-than-human’ care: Clients’ and counsellors’ views on the multiple affordances of chatbots in alcohol and other drug counselling | Peer reviewed journal article | Australia | Qualitative study | Yes, this paper identifies the range of possibilities for care that emerging chatbot interventions offer, including in relation to human care. | Yes, in-depth qualitative interviews elicit understanding of people’s thoughts on the care that chatbots can offer. | Yes | Yes |
| Bickmore | 2016 | Improving Access to Online Health Information with Conversational Agents: A Randomized Controlled Experiment | Peer reviewed journal article | USA | Randomised control experiment | Yes, this paper assesses the use of chatbot assisted searching compared to using traditional search engines**.** | Yes, a small randomised controlled experiment provides initial understanding of how chatbots can support users with web-based searches. | Yes | Yes |
| Bickmore | 2020 | Promotion of Preconception Care Among Adolescents and Young Adults by Conversational Agent | Peer reviewed journal article | USA | Secondary analysis of randomised control trial | Yes. The study seeks to understand the acceptability and feasibility of conversational agent use for promotion of preconception care. | Yes. Secondary analysis of intervention group participants experiences of using the conversational agent is appropriate for understanding feasibility and acceptability. | Yes | Yes |
| Brannock | 2019 | Investigating Attitudes and Preferences Towards a Chatbot Pregnancy Guide Within Facebook's Social Media Platform Amongst Pregnant Women in Kenya | Gray Literature: Thesis | Kenya | Development report | Yes. The study seeks to describe the development of a chatbot to support pregnant women and reports on qualitative user feedback. | Yes, development adequately described and appropriate qualitative data collection methods used to collect user feedback. | Yes | Yes |
| Bonnevie | 2021 | Layla's Got You: Developing a tailored contraception chatbot for Black and Hispanic young women | Peer reviewed journal article | USA | Development Report | Yes. A report on the design and development of a chatbot to provide contraceptive information. | Yes. User-centred design, user testing and design and development activities reported on. | Yes | Yes |
| Chinkam | 2021 | The Perspectives of Women and Their Health-Care Providers Regarding Using an ECA to Support Mode of Birth Decisions | Peer reviewed journal article | USA | Qualitative study | Yes. The study presents the perspectives of women and health care providers to assess the acceptability and feasibility of using a chatbot to support mode of birth decision. | Yes. Data from focus groups carried out with women and health care providers is appropriately analysed to ascertain acceptability and feasibility. | Yes | Yes |
| Chernick | 2021 | A User-Informed, Theory-Based Pregnancy Prevention Intervention for Adolescents in the Emergency Department: A Prospective Cohort Study | Peer reviewed journal article | USA | Cohort Study | Yes. This study reports on the efficacy of a chatbot for encouraging contraception uptake amongst adolescent girls. | Somewhat, a cohort study design allows for the efficacy of the chatbot in encouraging contraception uptake to be measured. However, it should be noted that the sample size it small (n=42) and there is no control group. | Yes | Yes |
| Cho | 2022 | Chatbot-mediated learning of cardiac auscultation | Peer reviewed journal article | Australia | Mixed methods pilot study | Somewhat, this study explores how chatbots convey information and may increase understanding in the context of teaching medical students about cardiac auscultation. | Yes, a mixed methods pilot study allows for an initial exploration of how chatbots may convey information for better understanding. | Yes | Yes |
| Curtis | 2020 | Improving User Experience of Virtual Health Assistants: Scoping Review | Peer reviewed journal article | N/A | Scoping review | Yes, this review explored user experiences of virtual health assistants or chatbots. | Yes, a scoping review collates chatbot user experience data to understand how to improve users’ experiences of chatbots for health. | Yes | Yes |
| De Cicco | 2020 | Millennials’ attitude toward  chatbots: an experimental study in  a social relationship perspective | Peer reviewed journal article | Italy | Qualitative survey study | Yes, this study attempts to understand young people’s perceptions of chatbots, particularly their social aspects. | Yes, a survey collects young peoples perceptions of chatbots. | Yes | Yes |
| Dhinagharan | 2022 | Designing, Developing, Evaluating, and Implementing a Smartphone-Delivered, Rule-Based Conversational Agent (DISCOVER): Development of a Conceptual Framework | Peer reviewed journal article | N/A | Development report | Somewhat, this paper reports on the development of a conceptual framework and the design and development of a chatbot for use in a health care setting. | Yes, design and development activities reported thoroughly. | Yes | Yes |
| Edwards | 2013 | Use of an interactive computer agent to support breastfeeding | Peer reviewed journal article | USA | Pilot feasibility trial | Yes. The study seeks to understand the feasibility and initial efficacy of a conversational agent to support breastfeeding | Yes. Well-designed pilot RCT methodology with an appropriate sample size for a pilot study. Intent, attitudes and confidence towards breastfeeding were measured, alongside satisfaction with the conversational agent. | Yes | Yes |
| Green | 2022 | Predicting healthcare-seeking behavior based on stated readiness to act: Development and validation of a prediction model. | Peer reviewed journal article | Kenya | Model development report | Yes. The model developed in this paper aims to predict whether chatbot users, who engage with the chatbot regarding contraceptives, will go on to access contraceptive services. | Yes. Model development is described in detail and model testing is appropriate. However, the model is not externally validated. | Yes | Yes |
| Garrett | 2022 | Potential application of conversational agents in HIV testing uptake among high-risk populations | Peer reviewed journal article | N/A | Narrative review | Yes. This well referenced paper presents the potential application of conversational agents for HIV testing uptake. | N/A | Yes | Yes |
| Handforth and Bertermann | 2018 | How Girl Effect built a chatbot | Technical report | South Africa | Development report | Yes. This technical report details the design and development of a chatbot aiming to increase SRJ knowledge and access efficacy and presents key learning. | Yes. User-centred design, user testing and design and development activities reported on. | Yes | Yes |
| Harrington | 2019 | Improving Access to Sexual Health Information with AI Chatbots | Website article | Kenya | N/A | Yes. This article discusses the aims, design, development and implementation of a chatbot aiming to provide SRH information. | N/A | Yes | Yes |
| Hassani | 2022 | Potential Role of Conversational Agents in Encouraging PrEP Uptake | Peer reviewed journal article | N/A | Narrative review | Yes. This well referenced paper presents the potential application of conversational agents for PrEP uptake. | N/A | Yes | Yes |
| Hussain | 2019 | Mobile Phone-Based Chatbot for Family Planning and Contraceptive Information | Gray literature: Conference paper | USA | Feasibility study | Yes. This paper reports on the feasibility and acceptability of a chatbot that disseminates contraceptive information to heterosexual couples. | Yes. The paper uses the UTAUT survey to measure feasibility and acceptability, including measures of performance expectancy, attitudes, effort expectancy, social influence, anxiety, behavioural intention, self-efficacy. | Yes | Yes |
| IPAS | 2021 | Got an abortion question? Ask Nurse Nisa | Website article | Kenya and the Democratic Republic of the Congo | N/A | Yes. This short article reports aims and some key learning from the developers of an abortion information dissemination chatbot. | N/A | Yes | Yes |
| Jack | 2020 | Promotion of Preconception Care Among Adolescents and Young Adults by Conversational Agent | Peer reviewed journal article | USA | Randomised Control Trial | Yes. RCT assessing the efficacy of a conversational agent to change preconception care behaviours. | Yes. Well-designed RCT methodology with a large sample size. The primary outcome was the proportion of identified risks at the action or maintenance stage of change. | Yes | Yes |
| Jack and Bickmore | 2009 | Taking the time to care: empowering low health literacy hospital patients with virtual nurse agents | Gray literature: Conference paper | USA | Development report | Yes, this development report describes the development of a chatbot aiming to educate patients on their care after hospital discharge and initial testing suggests that chatbots are helpful for those with low literacy. | Yes, development methodology, iterations and initial user testing reported on. | Yes | Yes |
| Kobori | 2018 | Novel application for sexual transmitted infection screening with an AI chatbot | Peer reviewed journal article | Japan | Usability and efficacy study | Somewhat. This study aims to understand the usability and efficacy of a chatbot that aims to accurately diagnose STIs. | Yes. The chatbots efficacy is appropriately tested in terms of accuracy of diagnosis. The sample is drawn from men presenting at a STI clinic so may not represent those that have limited access to services. | Yes | Yes |
| Lok Woo | 2020 | Development of Conversational Artificial Intelligence for Pandemic Healthcare Query Support | Peer reviewed journal article | UK | Development report | Yes. This paper describes the development process and user feedback on a chatbot designed to answer healthcare queries, including SRH queries, in a pandemic setting. | Yes. The development process is described in detail and user feedback is collected appropriately using surveys. | Yes | Yes |
| Lucas | 2014 | It’s only a computer: Virtual humans increase willingness to disclose | Peer reviewed journal article | USA | Observational study | Yes, this paper assesses whether people are more likely to disclose health information to humans or chatbots. | Yes, participants randomly interacted with a human or a chatbot and completed surveys to understand their experience of disclosing health information. | Yes | Yes |
| Maeda | 2020 | Promoting fertility awareness and preconception health using a chatbot: a randomized controlled trial | Peer reviewed journal article | Japan | RCT | Yes. This RCT assess the efficacy of a chatbot for improving preconception health, including oral contraception use. | Yes. A robust RCT methodology with a large sample size (n=927) assesses  chatbot efficacy. | Yes | Yes |
| Marcus | 2020 | Artificial Intelligence and Machine Learning for HIV Prevention: Emerging Approaches to Ending the Epidemic | Peer reviewed journal article | N/A | Narrative review | Somewhat. This review collates AI and Machine learning applications for HIV prevention including chatbots. | N/A | Yes | Yes |
| Nadarsynski | 2018 | The acceptability of artificial intelligence (AI)-led chatbot, an automated advice system for sexual health | Peer reviewed journal article | UK | Cross-sectional survey study | Yes. This study assesses the acceptability of chatbots disseminating sexual health advise. | Yes. A voluntary survey of attendees as a sexual health clinic on attitudes towards digital services is an appropriate methodology to assess acceptability of chatbots for SRH. This methodology cannot ascertain acceptability of chatbots with lack of access to SRH services or lack of digital access. | Yes | Yes |
| Nadarsynski | 2020 | Acceptability of artificial intelligence (AI)-enabled chatbots, video consultations and live webchats as online platforms for sexual health advice | Peer reviewed journal article | UK | Cross-sectional survey study | Somewhat. This study assesses the acceptability of digital SRH services including chatbots. | Yes. A voluntary survey of attendees as a sexual health clinic on attitudes towards digital services is an appropriate methodology to assess acceptability of digital SRH services. This methodology cannot ascertain acceptability of chatbots with lack of access to SRH services or lack of digital access. | Yes | Yes |
| Nadarsynski | 2021 | Barriers and facilitators to engagement with artificial intelligence (AI)-based chatbots for sexual and reproductive health advice: A qualitative analysis | Peer reviewed journal article | UK | Qualitative interview study | Yes. This study explores experiences of engagement with chatbots for SRH. | Yes. Collection of semi-structured interviews is an appropriate methodology for understanding the detail and depth of why people may or may not engage with chatbots for SRH. | Yes | Yes |
| Niben | 2022 | See you soon again, chatbot? A design taxonomy to characterize user-chatbot relationships with different time horizons | Peer reviewed journal article | N/A | Design taxonomy development | Yes, this paper reports on a design taxonomy which categorises chatbots with regards to how they engage with users over time. | Yes, well executed design taxonomy reports on useful categorisations of chatbots. | Yes | Yes |
| Oketch | 2022 | How a chatbot is disrupting stigma around sexual health | Website article | Kenya | N/A | Yes. This article discusses the aims, design, development and implementation of chatbots that disseminates SRH information. | N/A | Yes | Yes |
| Pickard | 2016 | Revealing sensitive information in personal interviews: Is self-disclosure easier with humans or avatars and under what conditions? | Peer reviewed journal article | USA | Exploratory study | Yes, this study aims to asses how people feel about disclosing sensitive information to humans versus chatbots. | Yes, surveys collect data from participants with regards to their feelings on disclosing different topics to chatbots or humans. | Yes | Yes |
| PSI | 2019 | Will access to sex-positive and reproductive health information through a chatbot lead to increased contraceptive use amongst Kenyan youth? | Website article | Kenya | Development report | Yes. This article reports on the design, development, implementation and evaluation of a pilot chatbot disseminating SRH and pleasure information. | Evaluation methods are constrained to numbers of users engaging with the chatbot and its different content areas. No statements can be made about the bots impact on SRH outcomes. | Yes | Yes |
| Rahman | 2021 | Adolescentbot: Understanding opportunities for chatbots in combating adolescent sexual and reproductive health problems in Bangladesh | Peer reviewed journal article | Bangladesh | Development report | Yes. Yes. This study reports on the design, development, implementation and initial user feedback of a chatbot aiming to disseminate SRH information. | Yes. The design and development process is described in detail and an online survey is appropriate for capturing initial user feedback. | Yes | Yes |
| Rapp | 2021 | The human side of human-chatbot interaction: A systematic literature review of ten years of research on text-based chatbots | Peer reviewed journal article | N/A | Literature review | Yes, this review explores the literature on chatbots with regards to human-chatbot interactions, including emotionality. | Yes, a well-executed review which reveals insights into human-chatbot relationships. | Yes | Yes |
| Say It Now | 2022 | A week in Uganda with Safe Hands and RHU | Website article | Uganda | N/A | Yes. This article discusses the aims, design and development of a chatbot aiming to disseminate SRH information. | N/A | Yes | Yes |
| Seering | 2019 | Beyond Dyadic Interactions: Considering Chatbots as Community Members | Gray literature: Conference paper | USA | Literature and commercial review | Yes, this review explores the literature and existing chatbots that go beyond dyadic relationships. | Yes, this review comprehensively collates the literature and available chatbots that go beyond dyadic relationships. | Yes | Yes |
| Seering | 2020 | It Takes a Village: Integrating an Adaptive Chatbot into an Online Gaming Community | Gray literature: Conference paper | USA | Observational study | Yes, this study observes an online community to which a chatbot is added, in terms of the chatbots impact on the interactions within the group. | Yes, the community-chatbot dynamic is reported on and analyses. | Yes | Yes |
| She the People | 2022 | Doctor Didi is a sexual health and reproductive right based Whatsapp chat bot for the community of women | Website article | India | N/A | Yes. This article discusses the aims of a SRH and rights chatbot. | N/A | Yes | Yes |
| Shops Plus | 2022 | HelloJubi answers questions and dispels myths on health topics | Website article | India | N/A | Yes. This article briefly discusses the aims, development and implementation of a chatbot aiming to disseminate SRH information. | N/A | Yes | Yes |
| Surgo Ventures | 2020 | Can a Private Chatbot Help Couples with the Sensitive Topic of Family Planning? | Website article | India | N/A | Yes. This article briefly discusses an approach for evaluating AskNivi to provide SRH information and linkage to services for couples. | N/A | Yes | Yes |
| UNFPA | 2022 | “I didn’t have someone to consult when I became pregnant” | Website article | Mongolia | N/A | Yes. This article presents a case study of the experiences of a young woman who has used a chatbot that aims to disseminate SRH information. | N/A | Yes | Yes |
| Wang | 2021 | CASS: Towards Building a Social-Support Chatbot for Online Health Community | Peer reviewed journal article | N/A | Development report | Yes, this paper reports on the design and development of a chatbot developed to interact with online health communities. | Yes, the design and development of this extra-dyadic chatbot is well documented. | Yes | Yes |
| Wang | 2022 | An Artificial Intelligence Chatbot for Young People's Sexual and Reproductive Health in India (SnehAI): Instrumental Case Study | Peer reviewed journal article | India | Instrumental case study | Yes. This paper describes the design, development and implementation of a SRH information dissemination chatbot. This paper also provides some qualitative user feedback insights. | Yes. A case study approach is appropriate to understand the process of development and implementation. Qualitative techniques are appropriately used to understand user experience. | Yes | Yes |
| Wasson | 2021 | Sexual reproductive health chatbots: should we be so quick to throw artificial intelligence out with the bathwater? | Peer reviewed journal article | N/A | Letter | Yes. This brief letter responds to Nadarzynski et al’s findings on the acceptability of chatbots for SRH. | N/A | Yes | Yes |
| Who’s On | 2022 | Why your standalone chatbot is failing | Website article | N/A | N/A | Yes, this article reports on the benefits of connecting chatbot to a wider service network, in a customer service context. | N/A | Yes | Yes |
| Wilson | 2017 | In bed with Siri and Google Assistant: a comparison of sexual health advice | Gray literature: Short report | Not context specific | Short report | Yes. This short report observes the differences in answers to SRH queries between voice assistant chatbots Google Assistant and Alexa, and Google searches. | Yes. Voice assistant chatbots and google search are asked a set of predetermined questions and their answers are compared for accuracy and relevance. Appropriate methodology for a short report. | Yes | Yes |
| Wilson | 2022 | The Development and Use of Chatbots in Public Health: Scoping Review | Peer reviewed literature | N/A | Scoping review | Yes, this scoping review explores the development and use of chatbots in public health. | Yes, a well-executed and comprehensive scoping review. | Yes | Yes |
| Winskell | 2021 | Building a Chatbot for Health Content? This is for you. | Website article | Nigeria and Uganda | N/A | Yes. The article presents key learning from the design, development and implementation of a chatbot providing SRH information. | N/A | Yes | Yes |
| Work & Co | 2022 | Planned Parenthood Case Study | Website article | USA | Development report | Yes. The article describes the design, development and implementation of a chatbot for SRH information dissemination. | Yes. A case study approach allows the design, development and implementation to be reported on in detail and depth. | Yes | Yes |
| Yadav | 2019 | Feedpal: Understanding Opportunities for Chatbots in Breastfeeding Education of Women in India | Peer reviewed journal article | India | Development Report | Yes. An in depth report on the design and development of a chatbot for information giving to breastfeeding mothers. | Yes. User-centred design, user testing and feedback reported in detail. Qualitative data collection situates chatbot use in an Indian context. | Yes | Yes |
| YLabs | 2022 | AskDoki: Healthcare WhatsApp Chatbot | Website article | Kenya | N/A | Yes. The article presents key learning from the design, development and implementation of a chatbot providing SRH information. | N/A | Yes | Yes |
| Yokotani | 2018 | Advantages of virtual agents over clinical psychologists during comprehensive mental health interviews using a mixed methods design | Peer reviewed journal article | Japan | Observational study | Yes, this study examines how participants respond and disclose information, including information on sex and sexuality, to humans versus chatbots in a mental health setting. | Yes, participants experiences of mental health interviews with chatbots and humans are observed and analysed. | Yes | Yes |
| You | 2020 | Facilitators and barriers to incorporating digital technologies into HIV care among cisgender female sex workers living with HIV in South Africa. | Peer reviewed journal article | South Africa | Qualitative focus group study | Somewhat. The paper seeks to understand female sex workers experiences of digital technologies for HIV prevention and care, including chatbots. | Yes. A focus group data collection methodology is appropriate for understanding experiences of digital technologies for SRH. | Yes | Yes |
| Zamani | 2023 | Conversational Information Seeking, An Introduction to Conversational Search, Recommendation, and Question Answering | Peer reviewed journal article | N/A | N/A | Yes, this text defines and presents applications, interactions, interfaces, design, implementation, and evaluation of conversational information seeking such as chatbots. | N/A | Yes | Yes |
